# Distribution pattern and habitat preference for *Lobelia* species (Campanulaceae) in five countries of East Africa

**DOI:** 10.3897/phytokeys.159.54341

**Published:** 2020-09-04

**Authors:** John K. Muchuku, Andrew W. Gichira, Shu-Ying Zhao, Jin-Ming Chen, Ling-Yun Chen, Qing-Feng Wang

**Affiliations:** 1 Key Laboratory of Plant Germplasm Enhancement and Specialty Agriculture, Wuhan Botanical Garden, Chinese Academy of Sciences, Wuhan 430074, China Wuhan Botanical Garden, Chinese Academy of Sciences Wuhan China; 2 Department of Botany, Jomo Kenyatta University of Agriculture and Technology, Nairobi 62000-00200, Kenya Jomo Kenyatta University of Agriculture and Technology Nairobi Kenya; 3 Sino-Africa Joint Research Center, Chinese Academy of Sciences, Wuhan 430074, China Sino-Africa Joint Research Center, Chinese Academy of Sciences Wuhan China; 4 School of Pharmaceutical Sciences, South-Central University for Nationalities, Wuhan 430074, China South-Central University for Nationalities Wuhan China; 5 Department of TCMs Pharmaceuticals, School of Traditional Chinese Pharmacy, China Pharmaceutical University, Nanjing 211198, China China Pharmaceutical University Nanjing China

**Keywords:** East Africa, *Flora of Tropical East Africa*, habitat preferences, *
Lobelia
*, vegetation region

## Abstract

East Africa is one of the centres of distribution and diversity for *Lobelia* L. (Campanulaceae, sub-family Lobelioideae). *Lobelia* habitats in East Africa have been facing habitat fragmentation and loss, which are recognised as a major threat to biodiversity. However, previous plant conservation studies in East Africa only focused on protected areas and ignored unprotected areas. Future conservation strategies of plants, such as *Lobelia*, will depend on knowledge of their distribution patterns and habitat preference in East Africa. To understand the distribution pattern and the habitat preference of *Lobelia* in five countries (Kenya, Uganda, Tanzania, Rwanda and Burundi) of East Africa, we conducted a literature review in the seven major vegetation regions (afro-alpine, afro-montane forest, drier savannah, grasslands, wetter savannah, Zambezian woodland and semi-desert and desert). We also employed meander and patterned searches, which allowed greater opportunities for recording *Lobelia* species. Our results showed that the genus is distributed in all of the seven regions of the five countries with 54 taxa. The afro-montane forest region, with 41 taxa, is the richest in species diversity, followed by the Zambezian woodland region with 18 taxa. The semi-desert and desert region has the lowest number with only four taxa. The afro-alpine region has 15 taxa, although the region is the smallest by area. The herbaceous type was found in all regions, while the giant type has a clear preference for the afro-alpine and afro-montane forest regions. Future conservation for *Lobelia* should consider its habitat preference by, for example, focusing on the afro-alpine and afro-montane forest regions. This study will facilitate the setting of future conservation strategies for *Lobelia*.

## Introduction

Understanding species richness, habitat preferences and geographical distribution patterns is imperative in formulating conservation strategies. Assessment of species habitat and distribution patterns dates back to the late 18^th^ century (Forster et al. 1778). Johann Reinhold Forster, a naturalist, observed that there were species diversity and distribution gradients from the Equator to the Pole (Forster et al. 1778). Later, in 1805, Alexander von Humboldt (Humboldt 2005) suggested that there may be drivers that influenced the localisation, distribution and migration of plant species on Earth. Plants may demonstrate habitat preferences due to lower survival rates outside their preferred habitats (Comita et al. 2007). Consequently, plants are not uniformly distributed in a region (Brown 2014).

East Africa (EA) includes Kenya, Uganda, Tanzania, Rwanda, Burundi, Ethiopia, Somalia and South Sudan (Kalisa et al. 2019) and is a crucial biodiversity hotspot that is characterised by elevated plateaus and isolated mountains (Wang et al. 2020). The biomes in these EA countries were divided into altitudinal zones (Hedberg 1970), which have individual species microhabitats. The biomes were also divided into different regions including afro-alpine, afro-montane forest, drier savannah, grasslands, wetter savannah, zambezian woodland and semi-desert and desert regions (Kiage and Liu 2006). The distribution pattern for the EA species mainly depended on several factors, including geological events, elevation, edaphic factors (Stuart et al. 1990; Herron 2006), distance from the Equator, presence or absence of gene flow barriers, recent quaternary epoch, habitat preferences and anthropogenic activities (Hedberg 1954; Abdi 2013). For example, *Arabis alpina* L. (Brassicaceae) and *Turritis glabra* L. (Brassicaceae) have different habitat preferences. *Arabis alpina* has a preference for stream-banks and cliffs in an altitude of 2800–4800 m, while *T. glabra* has a preference for grasslands in an altitude of 920–2650 m (Agnew and Agnew 1994).

*Lobelia* L. (Campanulaceae, sub-family Lobelioideae) includes about 437 species. The genus is cosmopolitan, distributed in both temperate and tropical regions of Africa, America, Australia, New Zealand, Hawaii, Asia and other regions (Ruas et al. 2001; Lammers 2011; Antonelli 2008; Kokubugata et al. 2012). Africa is a species diversity centre for *Lobelia* species (lobelias), with 37% of all lobelias distributed in Africa (Kokubugata et al. 2012). *Lobelia* is well represented in EA, particularly in the early administrative divisions of Kenya (K1–7), Tanzania (T1–8), Uganda (U1–4), Rwanda and Burundi (Thulin 1984), where it inhabits most of the mountains and surrounding lowlands. The giant lobelias that are native to the higher altitude zones of the mountain groups in the EA form a conspicuous element of the flora.

Habitat loss and fragmentation are the major causes of biodiversity loss worldwide (Sukumaran and Jeeva 2008; Laurance 2010). *Lobelia* habitats in East Africa have been facing habitat fragmentation and loss (Kebede et al. 2007). Future conservation strategies of *Lobelia* in EA will depend on knowledge of their distribution patterns, habitat threats and habitat preferences. However, plant conservation studies in the EA only focused on protected areas and ignored unprotected areas (Eilu et al. 2007).

**Figure 1.**
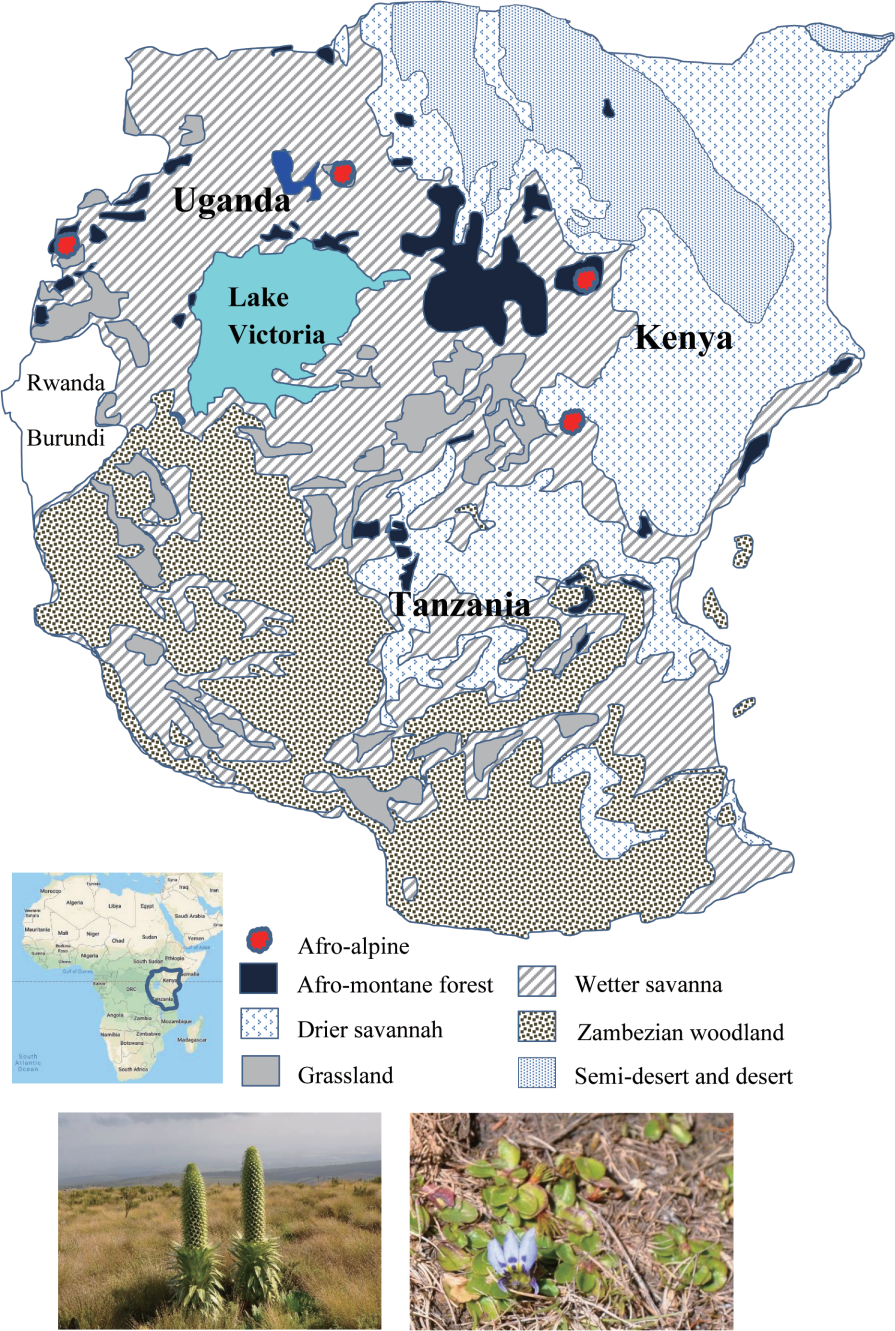
The major vegetation regions in East Africa, modified from Kiage and Liu (2006). Two pictures were used as examples of giant lobelias and herbaceous lobelias. The left picture is the giant *Lobelia deckenii* Hemsl. from Mt Kenya, while the right picture is the herbaceous *Lobelia lindblomii* Mildbr. from Mt Elgon (pictures by Ling-Yun Chen).

The distribution pattern of lobelias across different vegetations in the EA and the habitat preference remain unknown. As far as we know, lobelias are non-uniformly distributed in EA (Knox 1993; Kebede et al. 2007). For example, *Lobelia telekii* Schweinf. was only found on three mountains, Mt. Elgon, Aberdare Ranges and Mt. Kenya. Lobelia deckenii subsp. burtii (E.A.Bruce) Mabb., Lobelia burtii subsp. telmaticola E.B.Knox and Lobelia burtii subsp. meruensis E.B.Knox were only found in areas within the T2 region, specifically on Mt. Meru, Mt. Hanang and Mt. Loolmalassin in Tanzania (Thulin 1984). *Lobelia aberdarica* R.E.Fr. & T.C.E.Fr. inhabits the Cherangani Hills, Mau Ranges, Aberdare Ranges, Mt Elgon and Mt. Kenya. The upland afro-montane forest species *Lobelia giberroa* Hemsl. is widely spread within an altitude of 1200–3000 m in ten African countries including Kenya, Uganda, Tanzania, Sudan, Ethiopia, DR Congo, Rwanda, Burundi, Malawi and Zambia. The growth forms in this genus include annual or perennial herbs, shrubs or sub-shrub rosettes and small trees (Thulin 1984; Ruas et al. 2001). Amongst these growth forms, the most conspicuous are the branched inflorescence vs. unbranched inflorescence forms and giant vs. herbaceous forms (Fig. 1). Several studies have investigated the adaptation to afro-alpine environments, systematics and biogeography of lobelias (Knox and Palmer 1998; Kebede et al. 2007; Chen et al. 2016). For example, phylogeographic analyses indicated that *L. giberroa* could have migrated throughout different afro-mountains via the afro-montane forest bridge in previous interglacial periods (Kebede et al. 2007).

In this study, we reviewed previous literature and extracted data from our field survey. We aimed to identify the distribution pattern and habitat preference of *Lobelia* in five countries in the EA region (Fig. 1). This knowledge will facilitate the setting of future conservation strategies of the genus in EA.

## Materials and methods

### Study area

This study covered five of the eight East African countries, i.e. within and amongst the early administrative divisions of Kenya (K1–7), Uganda (U1–4), Tanzania (T1–8), Rwanda and Burundi. The other three countries (Ethiopia, Somalia and South Sudan) in EA have limited data from both herbaria and the *Flora of Tropical East Africa* (FTEA) on the distribution of the lobelias. Therefore, they were not included in this study. The study area ranged from coastal regions to the alpine zones of high mountains with changes in the elevation gradient. To obtain a clear understanding of the *Lobelia* habitats and distribution patterns, seven vegetation regions (afro-alpine, afro-montane forest, drier savannah, grasslands, wetter savannah, Zambezian woodland and semi-desert and desert) were used (Fig. 1; Kiage and Liu 2006). The vegetation regions in Rwanda and Burundi were not shown in the map of Fig. 1 (Kiage and Liu 2006). However, we recorded the distribution pattern of lobelias in the two countries by checking specimens and literature. Each lobelia in the two countries was assigned to the vegetation region, which is similar to the species’ habitat(s).

### Methods

All lobelias described in the *Flora of Tropical East Africa* (Thulin 1984), Knox and Pócs (1992) and Knox and Palmer (1998) were included in this study. The distribution and habitat for each species were explored using data from previous studies (Mabberley 1975; Thulin 1984; Knox and Pócs 1992; Agnew and Agnew 1994; Beentje et al. 1994; Knox and Palmer 1998; Knox et al. 2004; Zhou et al. 2017; Zhou et al. 2018), voucher specimens in the East African Herbarium of the National Museum of Kenya (NMK) and voucher specimens in the herbarium of Wuhan Botanical Garden (HIB) that had been collected from 2009 to 2019.

To maximise recording lobelias in traversed habitats, meander and patterned searches (Lancaster 2000) were used. The meander search was employed on difficult terrains, such as in mountains, deep river valleys and rocky hills. On the other hand, the systematic transect was employed on flat grounds, such as grassland and shrub-lands. Lobelias were identified to species or subspecies level.

## Results

The lobelias in the seven regions of the five countries are represented by 54 taxa including herbs, shrubs and sub-shrubs (Table 1).

**Table 1.** Lobelias diversity and distribution in Kenya, Uganda, Tanzania, Rwanda and Burundi of East Africa.

Species/subspecies name	Growth height	Elevation (m)	Habitat	Vegetation region	Data sources
*Lobelia aberdarica* R.E.Fr. & T.C.E.Fr.	Erect subshrub 3.5 m	1700–3550	Upland swamp	Amfr	SR, HS
*L. adnexa* E.Wimm.	Erect herb ca. 40 cm	1000–1600	Shady or rocky areas	Amfr, Zwr	FTEA
*L. angolensis* Engl. & Diels	Procumbent 25 cm	1600–2200	Moist wetland banks	Amfr, Zwr	SR, *FTEA*
*L. bambuseti* R.E.Fr. & T.C.E.Fr.	Erect subshrub 8 m	1800–3300	Forest, bamboo zone	Amfr	SR
*L. baumannii* Engl.	Procumbent herb 80 cm	700–2450	Stream banks in shade	Amfr, Gr, Wsr, Zwr	HS, *FTEA*
*L. bequaertii* De Wild.	Erect subshrub 4–5 m	3250–4100	Moorland and bog	A-ar	HS, *FTEA*
L. burttii subsp. meruensis E.B.Knox	Erect subshrub 3 m	3150–3900	Wet alpine or ravine	A-ar	SR, HS
L. burttii subsp. telmaticola E.B.Knox	Erect subshrub 3 m	3000–3900	Wet alpine and moorland	A-ar	SR, HS
*L. cheranganiensis* Thulin	Decumbent herb 0.6 m	2500–3400	Moorland	A-ar	SR
*L. chireensis* A.Rich.	Herb ca. 25 cm	500 –1250	Marshy muddy areas	Amfr, Dsr, Wsr, Zwr, S-ddr	*FTEA*
*L. cymbalarioides* Engl.	Prostrate herb ca. 70 cm	1500–3000	Moist forest and woodland floor	Amfr, Zwr	*FTEA*, LR
*L. deckenii* Hemsl.	Erect subshrub 4 m	3000–4500	Wet moorland	A-ar	SR
L. deckenii subsp. incipiens E.B.Knox	Erect subshrub 5 m	2700–3000	Mist forest	Amfr	SR
L. deckenii subsp. burtii (E.A.Bruce) Mabb.	Erect subshrub 3 m	3150–3800	Stream bank or ravine	A-ar	SR, HS, LR (Knox and Palmer 1998)
*L. dissecta* M.B.Moss	Erect herb ca. 50 cm	1500–2250	Open rocky area	Amfr	*FTEA*
*L. duriprati* T.C.E.Fr.	Decumbent herb 32 cm	1500–3600	Swamp or river banks	Amfr, Dsr, Zwr	SR
*L. erinus* L.	Decumbent herb ca. 65 cm	0–2500	Wet banks, grassland	Gr, Wsr, Zwr	SR, HS, *FTEA*
L. fervens subsp. recurvata (E.Wimm.) Thulin	Erect herb 60 cm	400–1500	Marshy areas, Savannah, forest	Amfr, Dsr, S-ddr	SR, *FTEA*
*L. fervens* Thunb.	Erect herb ca. 60 cm	10–2100	Grassland, forest and woodland edge, river banks	Amfr, Wsr, Zwr	SR, HS
L. flaccida subsp. granvikii (T.C.E.Fr.) Thulin	Erect herb 15–60 cm	1200–3200	Upland forest edges and on wet marshy edges	Amfr, Wsr, Dsr, Gr	SR, *FTEA*
*L. giberroa* Hemsl.	Erect shrub 10 m	1200–3050	Upland forest edges	Amfr	SR, LR (Kebede et al. 2007; Knox and Palmer 1998)
*L. gilgii* Engl.	Branched prostrate herb 45 cm	1500–2500	Stream banks	Amfr, Zwr	SR, *FTEA*
*L. goetzei* Diels	Erect herb 75 cm	1000–3000	Grassy rocky hillside	Amfr, Dsr, Gr, Wsr, Zwr	*FTEA*
*L. graniticola* E.Wimm.	Decumbent herb < 50 cm	2100–2500	Rocky slopes	Amfr	SR, *FTEA*
*L. gregoriana* Baker f.	Erect sub shrub 3 m	3200–4500	Erica zone, wet moorland	A-ar	SR
L. gregoriana subsp. elgonensis (R.E.Fr. & T.C.E.Fr.) E.B.Knox	Erect subshrub 2 m	3400–4100	Swamp or stream banks	A-ar	SR, *FTEA*
L. gregoriana subsp. satimae E.B.Knox	Erect subshrub 3 m	3300–4000	Wet moorland	A-ar	SR
*L. hartlaubii* Buchenau	Procumbent herb 90 cm	500–1300	River banks, forest	Amfr	*FTEA*
*L. heyneana* Schult.	Erect herb30 cm	1000–1800	Disturbed rocky area	Amfr, Zwr	*FTEA*
*L. holstii* Engl.	Erect/decumbent 60 cm	900–3500	Disturbed moorland, rocky and forest areas	A-ar, Amfr, Dsr, Gr, Wsr	SR
*L. inconspicua* A.Rich.	Erect herb ca. 20 cm	1000–2400	Ditches, woodland	Amfr, Dsr, Wsr, Zwr	SR, *FTEA*
*L. lindblomii* Mildbr.	Prostrate herb ca. 80 cm	3000–4300	Swampy or rocky places	A-ar	SR
*L. longisepala* Engl.	Erect subshrub 5 m	750–1500	Along the streams	Amfr	HS, *FTEA*
*L. lukwangulensis* Engl.	Erect subshrub 10 m	1700–2500	Forest edges	Amfr	HS, *FTEA*
*L. mildbraedii* Engl.	Erect subshrub 3.5 m.	1800–3050	Upland swamp	Amfr	HS, *FTEA*
*L. minutula* Engl.	Prostrate herb >70 cm	200–4000	Moorland, Forest	A-ar, Amfr	SR
*L. molleri* Henriques.	Decumbent herb 80 cm	850–2500	Upland shady and moist places	Amfr, Wsr, Gr	FTEA
*L. morogoroensis* E.B.Knox & Pócs	Erect subshrub 6 m	700–1400	Dry woodland, riparian forest	Amfr, Zwr	LR (Knox and Pócs 1992)
*L. neumannii* T.C.E.Fr.	Decumbent herb ca. 35 cm	1800–2800	Bare or rocky ground	Amfr, Gr	SR
*L. ovina* E.Wimm.	Erect herb 77 cm	1800–2500	Burnt forest	Amfr	HS, *FTEA*, SR
*L. petiolata* Hauman	Erect shrub 5 m	1900–2100	Moist forest	Amfr	*FTEA*, LR (Knox and Palmer 1998)
*L. ritabeaniana* E.B.Knox	Erect subshrub 6 m	2000–2250	Moist forest	Amfr	LR (Knox and Palmer 1998)
*L. rubescens* De Wild.	Decumbent 60 cm	700–3000	Bamboo zone, forest, woodland in wetland banks	Amfr, Zwr	*FTEA*
*L. sancta* Thulin	Erect subshrub 8 m	1900–2100	Mist summit forest	Amfr	HS, *FTEA*
*L. sapinii* De Wild.	Erect ca. 35 cm	400–1050	Woodland, grassland	Amfr, Wsr, Zwr	FTEA
*L. stricklandiae* Gilliland	Erect subshrub 6 m	1700–2000	Lowland forest to bamboo	Amfr	HS, *FTEA*
*L. stuhlmannii* Schweinf. ex Engl.	Erect subshrub 10 m	3000–4000	Afro-alpine region, moorland	A-ar	HS, *FTEA*
*L. telekii* Schweinf.ex Engl	Erect subshrub 4 m	3000–5000	Lower afro-alpine to snow line	A-ar	SR
*L. trullifolia* Hemsl.	Decumbent herb > 60 cm	1000–2750	Forest margins, rocky areas	Amfr, Zwr	SR, *FTEA*
L. trullifolia subsp. minor Thulin	Erect herb 15 ca. 60 cm	1050–2200	Rocky outcrop	Amfr, Dsr, S-ddr	SR, *FTEA*
*L. uliginosa* E.Wimm.	Erect 45 cm	1000–1800	Rocky forest/ bog	Amfr, Zwr	*FTEA*
*L. undzungwensis* Thulin	Erect shrub 9 m	1500–2400	Mist forest, rock outcrop	Amfr	LR (Knox et al. 2004)
*L. welwitschii* Engl. & Diels ex Diels	Erect herb 45 cm	400–3200	Wet banks, bogs, swamps	Amfr, Dsr, Gr, Wsr, Zwr, S-ddr	SR, *FTEA*
*L. wollastonii* Baker f.	Erect subshrub 7 m	3300–4400	Erica zone, moorland	A-ar	HS, *FTEA*

A-ar = Afro-alpine region, Amfr = Afro-montane forest region, Dsr = Drier savannah region, Gr = Grassland region, Wsr = Wetter savannah region, Zwr = Zambezian woodland region and S-ddr = Semi-desert and desert region. *FTEA* = *Flora of Tropical East Africa*, SR = Sight record, HS = Herbarium specimen, LR = Literature review; only if a species were not described in *FTEA*, literature was used to check the habitat and distribution.

(1) Afro-alpine region. In this study, the afro-alpine region includes the sub-alpine ericaceous zone and the afro-alpine zone. The sub-alpine ericaceous zone ranges from about 3000 m to 3800 m (Wesche et al. 2000) and is dominated by *Helichrysum* Mill., *Hypericum* L. and *Erica* Boehm. The afro-alpine region extends to over 4900 m and is dominated by giant species of *Dendrosenecio* (Hauman ex Hedberg) B.Nord. and *Lobelia*. The giant lobelias include *Lobelia stuhlmannii* Schweinf. & E.A.Bruce, *Lobelia deckenii* Hemsl., *Lobelia gregoriana* Baker f., Lobelia gregoriana subsp. satimae (R.E.Fr. & T.C.E.Fr.) E.B.Knox, *Lobelia burtii* E.A.Bruce, L. burtii subsp. telmaticola E.B.Knox, L. burtii subsp. meruensis E.B.Knox, *Lobelia bequaertii* (De Wild.) Mabb., *Lobelia wollastonii* Baker f. and *L. telekii*. These species are mainly distributed in the upper alpine zone (Tables 1, 2). Besides these, a few herbaceous species, including *Lobelia minutula* Engl., *Lobelia cheranganiensis* Thulin, *Lobelia holstii* Engl. and *Lobelia lindblomii* Mildbr. (Table 1; Thulin 1984), inhabit altitudes above 3000 m. Amongst the herbaceous species, *L. lindblomii* grows at the highest elevation (3000–4300 m), particularly in the upland grassland and moorland on Mt. Elgon (Kenya/Uganda), Aberdare Ranges and Mt. Kenya. Recurring fires on the ericaceous belt created a buffer zone between the lower afro-alpine and the upper afro-montane forest regions. This buffer zone was observed to provide regeneration habitats for the herbaceous species *L. minutula*, *L. lindblomii* and *L. holstii* (see Suppl. material 1: Table S1 for species details).

**Table 2.** Summary for the distribution of lobelias in the seven vegetation regions.

Vegetation region	Altitude (m)	Number of taxa	Number of giant taxa	Number of herbaceous taxa
Afro-alpine	3000–4900	15	11	4
Afro-montane forest	0–3000	41	13	28
Lowland montane forest	0–1500	12	3	9
Upland montane forest	1500–3000	29	10	19
Drier savannah	10–1000	9	0	9
Grassland	1200–3000	9	0	9
Wetter savannah	0–1250	11	0	11
Zambezian woodland	700–1500	18	1	17
Semi-desert and desert	400–1500	4	0	4

(2) Afro-montane forest region. The afro-montane forest region currently occurs in anthropogenically-fragmented patches in East Africa and has an altitudinal range of 0–3000 m (Thulin 1984). The lowlands of the montane (< 1500 m) include deciduous trees and shrubs species, such as *Celtis africana* Burm.f., *Senegalia* Raf. spps., *Vachellia* (wight & Arn.) Kuntze spps., *Ilex* L. spps., *Haplocoelum foliolosum* (Hiern) Bullock and *Ficus* L. spp., amongst others. These lowlands were well inhabited by nine herbaceous lobelias, which include *Lobelia erinus* L., Lobelia trullifolia subsp. minor Thulin, *Lobelia welwitschii* Engl. & Diels ex Diels, *Lobelia sapinii* De Wild., Lobelia fervens subsp. recurvata (E.Wimm.) Thulin, *Lobelia chireensis* A.Rich., *Lobelia inconspicua* A.Rich, *Lobelia adnexa* E.Wimm. and *Lobelia hartlaubi* Buchenau. The giant lobelias distributed in this vegetation include *Lobelia morogoroensis* E. B. Knox & Pócs, *Lobelia longisepala* Engl. and *L. giberroa* (Tables 1, 2) at altitudes ranging from 1200–1500 m (Thulin 1984). An interesting exception in this category is *L. giberroa*, which is widespread in Africa and has a distribution range that extends to about 3000 m (Table 1). This species inhabits a transition zone between the lowland and upland giant species. Interestingly, the lowland range of *L. giberroa* is found in understoreys of closed forest and riparian ecosystems, a similar habitat to its upland range. This provides insight into understanding the habitat preference of the species.

The highlands of the afro-montane forest region extend from 1500 m to 3000 m. These forests have recently been highly fragmented. They are similar in species composition across the East African countries. Dominant native species from other families found in this region include Olea europaea subsp. africana (Mill.) P.S. Green., *Juniperus procera* Hockst ex Endl., *Prunus africana* (Hook.) Kalkman., *Oldeania alpina* (K. Schum.) Stapleton and *Hagenia abyssinica* (Faber-Langendoen) J.F.Gmel. However, non-native species have become fully naturalised in disturbed areas for timber production. The species include *Grevillea robusta* A.Cunn. ex R.Br., *Casuarina equisetifolia* L., *Cupressus lusitanica* Lindl. Ex Parl., *Pinus patula* Schiede & Deppe ex Schltdl., *Eucalyptus globulus* Labill., *Eucalyptus saligna* Sm., *Corymbia citriodora* (Hook.) K.D.Hill & Johnson, *Corymbia maculata* (Hook.) K.D.Hill & Johnson, *Fraxinus pennsylvanica* f.colorata B.Boivin, *Araucaria cunninghamii* Sweet ex Courtois and *Acrocarpus fraxinifolius* Arn.

The afro-montane forest region above 1600 m is rich in lobelias. Most species at this altitude are herbaceous and include *Lobelia gilgii* Engl., *Lobelia graniticola* E.Wimm., *Lobelia trullifolia* Hemsl., *Lobelia uliginosa* E.Wimm., *Lobelia dissecta* M.B.Moss, *Lobelia neumannii* T.C.E.Fr., Lobelia flaccida subsp. granvikii (T.C.E.Fr.) Thulin, *Lobelia molleri* Henriques., *Lobelia rubescens* De Wild., *Lobelia heyniana* Spreng., *L. minutula*, *Lobelia cymbalarioides* Engl., *Lobelia duriprati* T.C.E.Fr., *L. holstii*, *Lobelia goetzei* Diels, *Lobelia ovina* E.Wimm., *Lobelia baumannii* Engl., *Lobelia fervens* Thunb. and *Lobelia angolensis* Engl. & Diels (Table 1).

The giant lobelias in this zone include *Lobelia mildbraedii* Engl., *Lobelia sancta* Thulin, *Lobelia stricklandae* Gilliland, *Lobelia lukwangulensis* Engl., *Lobelia ritabeaniana* E.B.Knox, Lobelia deckenii subsp. incipiens E.B.Knox, *Lobelia petiolata* Hauman, *Lobelia udzungwensis* Thulin, *L. aberdarica* and *L. bambuseti* (Thulin 1984; see Suppl. materials 1: Table S2 for species details).

(3) Drier savannah region. The drier savannah region is found in Kenya, Ethiopia and Somalia, where *Senegalia* Raf. spp., *Vachellia* Wight & Arn. spp. and *Commiphora* Jacq. spp. dominate the area. The region ranges from 10 m to 1000 m (or up to 1500 m in some habitats; Thulin 1984). Herbaceous lobelias are found in muddy ditches, river or marshy edges and seasonally-flooding grasslands. These species include L. fervens subsp. recurvata, *L. goetzei*, *L. duriprati*, *L. inconspicua*, L. flaccida subsp. granvikii, L. trullifolia subsp. minor, *L. chireensis*, *L. holstii* and *L. welwitschii* (Table 1). No giant lobelias were found in this region (see Suppl. material 1: Table S3 for species details).

(4) Grassland region. Grassland region is the most common habitat in the EA, and is dominated by alternating grasses with thorny bush-land and thicket. Although habitats in this region are different from others, some lobelias in this region are the same as in the areas of highland, wet and dry savannah and some even from the woodland. However, lobelias in this region are mostly restricted to the wet ground after rainfall (waterlogged grassland and seasonal river banks) and at the edges of wetlands in the marshy areas, streams and river banks, as well as tops of grass hills. The species include *L. fervens*, *L. molleri*, *L. holstii*, L. flaccida subsp. granvikii, *L. baumannii*, *L. goetzei*, *L. erinus* and *L. welwitschii* (Table 1). No giant lobelias were found in this region (see Suppl. material 1: Table S4 for species details).

(5) Wetter savannah region. This region is widely distributed in Kenya, Tanzania and Uganda with altitudes from 0 m to 1250 m. Both Combretaceae R.Br., and Fabaceae Lindl. families dominate this region. Lobelias in this region include the herbaceous L. fervens subsp. fervens, *L. baumannii*, *L. goetzei*, *L. inconspicua*, *L. molleri*, *L. holstii*, L. flaccida subsp. granvikii, *L. sapinii*, *L. chireensis*, *L. erinus* and *L. welwitschii* (Table 1). No giant lobelias were found in this region (see Suppl. material 1: Table S5 for species details).

(6) Zambezian woodland region. This region occurs in the southern part of Tanzania at altitudes ranging from 700 m to 1500 m. It is dominated by members of the family Fabaceae, such as species of the genera *Brachystegia* Benth., *Julbernardia* Pellegr., *Isoberlinia* Craib & Stapf ex Holland and *Uapaca* Baill. *Lobelia* inhabits wet deciduous woodland habitats in this region. Herbaceous lobelias include L. fervens subsp. fervens, *L. chireensis*, L. trullifolia subsp. trullifolia, *L. gilgii*, *L. sapinii*, *L. duriprati*, *L. goetzei*, *L. inconspicua*, *L. uliginosa*, *L. rubescens*, *Lobelia heyneana* Schult., *L. adnexa*, *L. cymbalarioides*, *L. baumannii*, *L. erinus*, *L. angolensis* and *L. welwitschii* (Table 1). The only giant lobelia is *L. morogoroensis* (Knox and Pócs 1992), which is distributed near Morogoro, Tanzania at altitudes from 725–1400 m and may extend up to 2000 m (see Suppl. material 1: Table S6 for species details).

(7) Semi-desert and desert region. The hostile climate of this region forms semi-desert and desert vegetations in northern Kenya and southern Ethiopia. This region is characterised by thorny scattered trees and shrubs. Lobelias are distributed in muddy ditches edges and seasonal river banks. However, they are exceedingly rare and only appear in the seasonally-flooded ground or marshy edges of the freshwater wetlands. Species that are distributed in this region include L. fervens subsp. recurvata, L. trullifolia subsp. minor, *L. welwitschii* and *L. chireensis* (Table 1) (see Suppl. material 1: Table S7 for species details).

## Discussion

### Distribution patterns for lobelias in five countries of East Africa

We employed a map (Fig. 1) with seven vegetation regions to cover the distribution of lobelias in Kenya, Uganda, Tanzania, Rwanda and Burundi (Kiage and Liu 2006). The seven regions are mainly dominated by stepped plateaus, flat land savannah, highlands, mountains and wetland ecosystems. The lobelias in the seven regions are represented by 47 species and seven subspecies including herbs, shrubs and sub-shrubs (Table 1). The most elevated habitat was inhabited by a giant species *L. telekii* on Mt. Kenya, while the lowest habitat was inhabited by herbaceous species, such as *L. fervens* and *L. erinus*. The altitudinal distribution of lobelias could have been shaped by their habitat preferences and by the adaptive evolution (Hedberg 1964; Chen et al. 2015). For example, *L. telekii* was confined to the afro-alpine belt characterised by an extreme climate with “summer every day and winter every night” (Hedberg 1964).

The EA Mountains form altitudinal island-like habitats. Most of these mountains, which are known as sky-islands, are of volcanic origin (Hedberg 1969) and lie within the Latitudes 2°N and 4°S. The mountain vegetation is completely different from that of the surrounding lowland. For example, the savannah and the highland forest are different from those of the upper alpine in both species richness and growth habits (Tables 1, 2). Amongst the five sub-floras of EA, Tanzania with 38 taxa is the richest in lobelias followed by Kenya (21), Uganda (19), Burundi (9) and Rwanda (8) (Suppl. material 1: Tables S8–S12).

### East African Mountains are a centre of endemism for giant lobelias

The East African Mountains embrace a wide range of altitudinal habitats and ecosystems, from surrounding environs at the foot of the mountains to the alpine zone (Zhou et al. 2018). The diversity and richness of lobelias can thus seem to be dependent on existing diverse habitats. In general, the diversity of giant lobelias varies greatly within habitats. The diversity is higher in the afro-montane forest region (13 taxa, Table 2), followed by the afro-alpine region (11 taxa). The giant lobelias demonstrated a clear preference for afro-montane forest and afro-alpine regions. On the other hand, the number of herbaceous lobelias is higher in the afro-montane forest (28 taxa) and Zambezian woodland regions (17 taxa). The herbaceous type demonstrates a clear preference for a habitat characterised by trees in both afro-montane forest and Zambezian woodland regions.

East African Mountain biomes were differentiated into two major categories, the mountains that reach the alpine (upland) and those without the alpine regions (lowlands and Eastern Arc Mountains). The upland giant lobelias, except *L. petiolata*, normally have a single aerial stem that is unbranched. The branched *L. petiolata* inhabits Nyungwe in Rwanda and extends its range to Kahuzi in DR Congo in very wet afro-montane forest region. These species have a clear preference for a wet forest habitat (Knox and Palmer 1998).

The Eastern Arc Mountains (lowlands) form the easternmost blocks of East Africa (Burgess et al. 2007). Although they do not extend to the afro-alpine region, their location creates a Massenerbung effect, therefore generating variable and unique habitats (Knox and Palmer 1998). The lowland biomes form habitats characterised by the branched lobelias (Knox and Palmer 1998). These species include *L. undzungwensis*, *L. morogoroensis*, *L. stricklandiae*, *L. ritabeaniana*, *L. sancta*, *L. lukwangulensis* and *L. longisepala*. The Arc Mountains have evolved their distinctive lobelias, which are different from those of the upland mountains. The Arc Mountain biome, therefore, is a hotspot for the East Africa branched lobelias. The regions inhabited by lobelias have moist and warm habitats somewhat differentiated from the regions occupied by the inland giant lobelias. The Arc Mountain giant lobelias demonstrated a clear preference for moist and warm conditions within the mountains along the East Africa Coast, which is known as one of the world’s biodiversity hotspots (Stuart et al. 1990).

The Eastern Arc Mountains are separated from each other by lowland woodlands and savannah (Conte 2010). The Arc Mountain lobelias are distributed from open sites in seasonally-dry, semi-deciduous woodland (*L. morogoroensis*) to the sub-montane rainforests openings (*L. longisepala*) and also extend further to the cloud-forest summits (*L. ritabeaniana*, *L. stricklandiae*, *L. undzungwensis*, *L. sancta* and *L. lukwangulensis* (Table 1; Knox and Palmer 1998). Surprisingly, *L. giberroa*, which is found in the inland afro-montane forest, also grows on these Arc Mountains. *Lobelia giberroa* in the Arc Mountains occupies habitats with similar physiognomic characteristics and similar associated plant species found elsewhere in the highland afro-montane forests (Knox and Palmer 1998). The Eastern Arc and other East African mountains demonstrated an extraordinary pattern of lobelias’ endemism and community preferences.

### Major identified threats to afro-mountain lobelias’ habitat and its lowlands environments

Land-use changes associated with deforestation and land degradation are major causes of biodiversity loss in East Africa (Maitima et al. 2009). Currently, the afro-montane forest is fragmented and remains isolated around the EA Mountains. Without the present anthropogenic activities, the afro-montane forest would probably have existed as a single connected habitat (Hedberg 1969). The habitat fragmentation threatens the distribution patterns of some native species, for example, *L. giberroa* (Kebede et al. 2007). Therefore, habitat loss and fragmentation could threaten the forest-dependent lobelias. For example, *L. bambuseti* has been reported as being under threat (Kipkoech et al. 2019).

Moreover, the afro-montane forest and its environment are also threatened by invasive plants (Obiri 2011; Hulme et al. 2013). Most of the mountains in the EA have become centres for ecotourism. To facilitate tourist activities, roads and paths within the mountain have been built. The invasive species could follow the roadsides to reach different altitudes (Pauchard et al. 2009). According to our observation, the disturbed roadsides created opportunities for disturbed habitat lobelias to dominate the roadsides. For example, *L. holstii* has expanded its range to almost 3900+ m on Mt. Kenya and dominated the roadsides in rocky disturbed places. Additionally, non-native tree species established in the forest plantation for timber production may pose a considerable threat (Pauchard et al. 2009) to native forest lobelias’ ecosystems.

Mountain forests are major water towers, biodiversity hotspots, species evolution refugia, eco-tourism locations, sources of wild foods and centres of plant genetics (Viviroli and Weingartner 2008; Moelg et al. 2013; Kanui et al. 2016; Immerzeel et al. 2020). For example, they serve as a source of clean water for the drier lowlands, which are inhabited by lobelias, as observed in our extensive field survey. The afro-montane forest also provides places of cultural practices, such as religion for the native communities. Although lobelias are widely distributed in the seven vegetation regions, habitat loss is a challenge that calls for action. For example, the increasing human population has threatened the EA Mountain biomes. The habitat preference of lobelias’ hotspots is also facing the risk of both anthropogenic and climate change (Chala et al. 2016). Habitat destruction and fragmentation are recognised as a major threat to biodiversity (Liao et al. 2013). Therefore, the afro-montane forest and its environs are especially in need of protection (Immerzeel et al. 2020).

## Conclusions

Our results showed that lobelias are distributed in all of the seven vegetation regions in five countries of East Africa. The afro-montane forest region is the richest in species diversity, although it is not the largest by area. The herbaceous type has a preference for the lowland regions, while the giant type has a clear preference for the afro-alpine and afro-montane forest. Future conservation for the genus should consider the habitat preferences of lobelias.

## Supplementary materials

Supplementary material 1 Tables S1–S12 Data type data statistics Explanation note Data statistics for each species. https://binary.pensoft.net/file/449503This dataset is made available under the Open Database License (http://opendatacommons.org/licenses/odbl/1.0/). The Open Database License (ODbL) is a license agreement intended to allow users to freely share, modify, and use this Dataset while maintaining this same freedom for others, provided that the original source and author(s) are credited.John K. Muchuku, Andrew W. Gichira, Shu-Ying Zhao, Jin-Ming Chen, Ling-Yun Chen, Qing-Feng Wang
