# Supplementary material for: Distribution pattern and habitat preference for Lobelia species (Campanulaceae) in five countries of East Africa
Source: PhytoKeys. 2020 Sep 4;159:45–60. doi: 10.3897/phytokeys.159.54341 (PMC7486314; doi:10.3897/phytokeys.159.54341)
Supplement: Supplementary material 1 — Tables S1–S12 [file phytokeys-159-045-s001.docx]

Table S1. Giant and herbaceous lobelias in the afro-alpine region. EA = East Africa. *FTEA* = *Flora of Tropical East Africa*

| **Taxa name** | **Habit / height** | **Elevation (m)** | **Habitat in EA** | ***FTEA* flora regions** |
| --- | --- | --- | --- | --- |
| *L. bequaertii* | Erect subshrub 4–5 m | 3250–4100 | Moorland and bog | U2 |
| *L. burtii* subsp. *meruensis* | Erect subshrub 3 m | 3150–3900 | Wet alpine moorland | T2 |
| *L. burttii* | Erect subshrub 3m | 3150–3800 | Stream bank or ravine | T2 |
| *L. burttii* subsp. *telmaticola* | Erect subshrub 3 m | 3000–3900 | Wet alpine moorland | T2 |
| *L. cheranganiensis* | Decumbent herb 60 cm | 3000–3400 | Lower moorland | K3 |
| *L. deckenii* | Erect subshrub 4 m | 3000–4500 | Moorland | T2 |
| *L. gregoriana* | Erect subshrub 3 m | 3200–4500 | Moorland | K4 |
| *L. gregoriana* subsp. *elgonensis* | Erect subshrub 2 m | 3400–4100 | Moorland near water | K3, U3 |
| *L. gregoriana* subsp. *sattimae* | Erect subshrub 3 m | 3300–4000 | Moorland | K3, 4 |
| *L. holstii* | Decumbent 60 cm | 3000–3400 | Disturbed moorland | K1–7, T2, 3, 5–6, Rwanda, Burundi |
| *L. lindblomii* | Prostrate herb 80 cm | 3000–4300 | Alpine swampy areas | U3, K3, 4 |
| *L. minutula* | Prostrate herb 70 cm | 3000–3300 | Moorland | U2, K 3, 4, 6, T2, 7, Rwanda, Burundi |
| *L. stuhlmanni* | Erect subshrub 10 m | 3000–4000 | Moorland | U2, Rwanda |
| *L. telekii* | Erect subshrub 4 m | 3000–5000 | Moorland to snow line | U3, K3, 4 |
| *L. wollastonii* | Erect subshrub 7 m | 3300–4000 | Moorland | U2, Rwanda |

Table S2. Giant and herbaceous lobelias in the afro-montane forest region.

| **Taxa name** | **Habit / height** | **Elevation (m)** | **Habitat in EA** | ***FTEA* Flora regions** |
| --- | --- | --- | --- | --- |
| *L. aberdarica* | Erect subshrub 3.5 m | 1700–3000 | Swamp and wet forest | K3–6, U3 |
| *L. adnexa* | Erect herb 40 cm | 1000–1500 | Shady forest areas | U4, K2–5, T2, 4, 7, 8 |
| *L. angolensis* | Procumbent herb 25 cm | 1500–2200 | Moist wetland banks | K3, T7 |
| *L. bambuseti* | Erect subshrub 8 m | 1800–3000 | Upper afro-montane forest | K3, 4 |
| *L. baumannii* | Procumbent herb 80 cm | 1500–2400 | Stream banks in forest | K4, T2–4, 6–8 |
| *L. chireensis* | Erect herb 25 cm | 500–1200 | Marshy, wet lowland, Forest grassland | T4, Burundi |
| *L. cymbalarioides* | Prostrate herb 70 cm | 1500–3000 | Wet forest floor | T2, 3, 6 |
| *L. deckenii* | Erect subshrub 5 m | 2700-3000 | Mist forest | T2 |
| *L. dissecta* | Erect herb 50 cm | 1500–2250 | Open rocky forest area | U1, Burundi |
| *L. duriprati* | Decumbent herb 32 cm | 1500–3000 | Swamp or river banks | T2, 3, 6 |
| *L. erinus* | Decumbent herb 65 cm | 0–1500 | Dry forest/ wet banks | T4, 7 |
| *L. fervens* | Erect herb 60 cm | 1500–1800 | Mid altitude, coastal upland forest edges area | K1, 4, 7, T1, 3, 4, 6–8 |
| *L. fervens* subsp. *recurvata* | Erect herb 60 cm | 400–1500 | Grassland, Dry forest wet banks | U2–4, K1, T1, 2, 5–7 |
| *L. flaccida* subsp. *granvikii* | Erect herb 15–60 cm | 1200–3100 | Upland forest edges and on marshy | U1, 3, 4, K3, 5, 6 |
| *L. giberroa* | Erect subshrub 9 m | 1200–3000 | Forest edges, Riparian forest | K1, 3–7, U1–3, T2–4, 6–8, Rwanda, Burundi |
| *L. gilgii* | Prostrate herb 45 cm | 1500–2500 | Stream banks | T6 |
| *L. goetzei* | Erect herb75 cm | 1500–3000 | Rocky forest hillside | T7, 8 |
| *L. graniticola* | Decumbent herb 50 cm | 2100–2500 | Rocky forest slopes | T6 |
| *L. hartlaubii* | Procumbent herb 90 cm | 500–1300 | River banks and forest | U2, 4 Rwanda, Burundi |
| *L. heyneana* | Erect herb 30 cm | 1500–1800 | Disturbed rocky area | T3 |
| *L. holstii* | Erect/decumbent herb 60 cm | 1500–3400 | Rocky and forest areas | K1–7, T2, 3, 5–6, Rwanda, Burundi |
| *L. inconspicua* | Erect herb 20 cm | 1000–1550m | Ditches and dry forest | U3, K3–6, T2 |
| *L. Longisepala* | Erect subshrub 5 m | 750–1500 | Forest stream banks | T3 |
| *L. Lukwangulensis* | Erect subshrub 10 m | 1700–2500 | Forest edges | T6 |
| *L. mildbraedii* | Erect subshrub 3.5 m | 1800–3000 | Upland swamp forest | Burundi |
| *L. minutula* | Prostrate herb 70 cm | 2000–3000 | Moorland and Forest | U2, K3, 4, 6, T2, 7, Rwanda, Burundi |
| *L. molleri* | Decumbent herb 80 cm | 1500–2500 | Upland forest edges, and shady moist places | U2–4, T6, Burundi |
| *L. morogorensis* | Erect subshrub 6 m | 700–1400 | Dry forest | T3 |
| *L. neumannii* | Decumbent herb 35 cm | 2100–2800 | Bare or rocky forest | K2–4 |
| *L. ovina* | Erect herb 77 cm | 1500–2500 | Burnt forest | T7, 8 |
| *L. petiolata* | Erect subshrub 5 m | 1900–2100 | Moist forest rocky or bare ground | Rwanda |
| *L. ritabeaniana* | Erect subshrub 6 m | 2000–2250 | Moist forest | T3 |
| *L. rubescens* | Decumbent herb 60 cm | 1500–3000 | Bamboo zone, forest, wetlands banks | U2, T6, Burundi, Rwanda |
| *L. Sancta* | Erect subshrub 8 m | 1900–2100 | Mist summit forest | T6 |
| *L. sapinii* | Erect herb 35 cm | 400–1050 | Forest grassland | T6 |
| *L. stricklandiae* | Erect subshrub 6 m | 1700–2000 | Forest to bamboo | T4 |
| *L. trullifolia* | Decumbent herb 60 cm | 1500–2700 | Forest margins and often | K2, T2–3, 6–8 |
| *L. trullifolia* subsp. *minor* | Erect herb 15–60 cm | 1050–1500 | Rocky forest outcrop | T7, 8 |
| *L. uluginosa* | Erect herb 45 cm | 1500–1800 | Rocky forest/ bog | T4 |
| *L. undzungwensis* | Erect subshrub 3 m | 1500–2400 | Mist forest | T4 |
| *L. welwitschii* | Erect herb 45cm | 400–1500 | Wet banks, bogs and swamps | U1, K3, 5, T4, 7, 8 |

Table S3. Herbaceous lobelias in the drier savannah region.

| **Taxa name** | **Habit / height** | **Elevation (m)** | **Habitat in EA** | ***FTEA* Flora regions** |
| --- | --- | --- | --- | --- |
| *L. chireensis* | Erect herb 25 cm | 500–1500 | Marshy, wet river banks | T4, Burundi |
| *L. duriprati* | Decumbent herb 32 cm | 900–1500 | Wet water banks | U3, K3–6, T2 |
| *L. fervens* subsp. *recurvata* | Erect herb 60 cm | 400–1500 | Savannah grass land stream side, wet woodland | U2–4, T1, 2, 5–7, Burundi |
| *L. flaccida* subsp. *granvikii* | Erect herb15–60 cm | 1200–1500 | Midland wet grounds and river banks | U1, 3, 4, K3, 5, 6 |
| *L. goetzei* | Erect herb 75 cm | 1000–1500 | Wet Savannah and hillside | T7, 8. |
| *L. holstii* | Erect herb 60 cm | 900–1500 | Disturbed marshy area | K1–7, T2, 3, 5–6, Rwanda, Burundi |
| *L. inconspicua* | Erect herb20 cm | 1000–1500 | Wet Savannah grass land | U3, K3–6, T2 |
| *L. trullifolia* subsp. *minor* | Erect herb15–60 cm | 1100–1500 | Rocky river banks | T7, 8 |
| *L. welwitschii* | Erect herb 45 cm | 400–1500 | Wet banks and swamps | U1, K 3, 5, T4, 7, 8 |

Table S4. Herbaceous lobelias in the grassland region.

| **Taxa name** | **Habit / height** | **Elevation (m)** | **Habitat in EA** | ***FTEA* Flora regions** |
| --- | --- | --- | --- | --- |
| *L. baumannii* | Procumbent herb 80 cm | 1500–2400 | Upland wet grassland | K4, T2–4, 6–8 |
| *L. erinus* | Decumbent herb 65 cm | 1500–2500 | Wet grassland | T4, 7 |
| *L. fervens* | Erect herb 60 cm | 1500–1200 | Mid altitude wet grassland | K1, 4, 7, T1, 3, 4, 6–8 |
| *L. flaccida* subsp. *granvikii* | Erect herb 15–60 cm | 1200–2700 | Wet upland wooded grassland | U3, 4, K3, 5,6 |
| *L. goetzei* | Erect herb 75 cm | 1700–3000 | Wet grassland hillside | T7, 8 |
| *L. holstii* | Erect/decumbent herb 60 cm | 2000–2700 | Wet Sloppy grassland | K1–7, T2, 3, 5–6 |
| *L. molleri* | Decumbent herb 80 cm | 2000–2500 | Wet upland grassland | U3–4, T6, Burundi |
| *L. neumannii* | Decumbent herb 35 cm | 2100–2800 | Wet wooded grassland | K2–4 |
| *L. welwitschii* | Erect 45 cm | 1500–2800 | Midland wet grassland | U1, K3, 5, T4, 7 |

Table S5. Herbaceous lobelias in the wetter savannah region.

| **Taxa name** | **Habit / height** | **Elevation (m)** | **Habitat in EA** | ***FTEA* Flora regions** |
| --- | --- | --- | --- | --- |
| *L. baumannii* | Procumbent herb 80 cm | 600–2000 | Stream banks in shade | K4, T2–4, 6–8 |
| *L. chireensis* | Herb 25 cm | 500–1500 | Wet midland and river banks | T4, Burundi |
| *L. erinus* | Decumbent herb 65 cm | 1500–2450 | Wet river banks | T4, 7 |
| *L. fervens* subsp. *fervens* | Erect herb 60 cm | 600–2000 | Savannah riparian forest edge and wet soil | K1, 4, 7, T1, 3, 4, 6–8 |
| *L. flaccida* subsp. *granvikii* | Erect herb 15–60 cm | 1500–26000 | Midland savannah riparian forest edges and river wet banks | U1, 3, 4, K3, 5, 6 |
| *L. goetzei* | Erect herb 75 cm | 1000–2000 | Savannah riparian stream banks | T7, 8 |
| *L. holstii* | Erect herb 60 cm | 1500–2500 | Disturbed marshy area rocky and savannah riparian forest areas | K1–7, T2, 3, 5–6 |
| *L. inconspicua* | Erect herb 20 cm | 1000–1500 | Wet savannah grass land | U3, K3–6, T2 |
| *L. molleri* | Decumbent herb 80 cm | 850–2300 | Upland savannah riparian Shady and Moist savannah ditches banks | U2–4, T6, Burundi |
| *L. sapinii* | Erect herb35 cm | 400–1050 | Wooded wet grassland | T6 |
| *L. welwitschii* | Erect herb 45 cm | 400–1500 | Wet savannah riparian banks and swamps | U1, K3, 5, T4, 7, 8 |

Table S6. Lobelias in the Zambezian woodland region.

| **Taxa name** | **Habit / height** | **Elevation (m)** | **Habitat in EA** | ***FTEA* Flora regions** |
| --- | --- | --- | --- | --- |
| *L. adnexa* | Erect herb 40 cm | 1000–1600 | Shady or rocky areas | U4, K2–5, T2; 4, 7, 8 |
| *L. angolensis* | Procumbent herb 25 cm | 1500–2000 | Moist wetland banks | K3, T7 |
| *L. baumannii* | Procumbent herb 80 cm | 600–2400 | Stream banks in shade | K4, T2–4, 6–8 |
| *L. chireensis* | Herb 25 cm | 300–1200 | Marshy and flooding grassland | T4, Burundi |
| *L. cymbalarioides* | Prostrate herb 70 cm | 1500 | Wet woodland floor | T2, 3, 6 |
| *L. duriprati* | Decumbent herb 32 cm | 1500–2100 | Swamp or river banks | U3, K3–6, T2 |
| *L. erinus* | Decumbent herb 65 cm | 0–2000 | Wet banks, and grassland | T4, 7 |
| *L. fervens* subsp. *fervens* | Erect herb 60 cm | 0–1200 | Coastal strip wet woodland and river inlet | K1, 4, 7, T1, 3, 4, 6–8 |
| *L. gilgii* | Prostrate herb45 cm | 1500 | Wet stream banks | T6 |
| *L. goetzei* | Erect herb 75 cm | 1000–2000 | Grassy wet rocky hillside | T7, 8. |
| *L. heyneana* | Erect herb 30 cm | 1000–1800 | Disturbed rocky area | T3 |
| *L. inconspicua* | Erect herb 20 cm | 1000–1500 | Woodland wet grassland | U3, K3–6, T2 |
| *L. morogorensis* | Erect subshrub 6 m | 700–1200 | Dry and riparian woodland | T3 |
| *L. rubescens* | Decumbent herb 60 cm | 700–1500 | woodland, wetlands banks | U2, T6, Burundi |
| *L. sapinii* | Erect herb 35 cm | 400–1050 | Woodland in wet grassland open areas | T6 |
| *L. trullifolia* subsp. *trullifolia* | Decumbent herb 60 cm | 1000–1500 | woodland edges and often rocky or bare ground | K2, T2–3, 6–8 |
| *L. uluginosa* | Erect herb 45 cm | 1000–1800 | Rocky bog and woodland | T4 |
| *L. welwitschii* | Erect herb 45 cm | 400–1500 | Wet banks and swamps | U1, K3, 5, T4, 7, 8 |

Table S7. Lobelias in the semi-desert and desert region.

| **Taxa name** | **Habit / height** | **Elevation (m)** | **Habitat in EA** | ***FTEA* Flora regions** |
| --- | --- | --- | --- | --- |
| *L. chireensis* | Herb 25 cm | 500–1200 | River banks, wet grassland | T4, Burundi |
| *L. fervens* subsp. *recurvata* | Erect herb 60 cm | 400–1500 | Flooding grassland Marshy areas | U2–4, K, T1, 2, 5–7, Burundi. |
| *L. trullifolia* subsp. *minor* | Erect herb 15–60 cm | 1050–1500 | Wet rocky outcrop | T7, 8 |
| *L.welwitschii* | Erect herb 45 cm | 400–1500 | Wet ditches banks, and swamps | U1, K 3, 5, T4, 7, 8 |

Table S8. Lobelias and altitudinal distribution in the Tanzania’s Flora regions (T1–8 and including Zanzibar (Z) a sub-flora of the *Flora of Tropical East Africa*.

| **Taxa name** | **Elevation in the region (m)** | **Early administrative Flora regions (T1-8) and Zanzibar island (Z)** |
| --- | --- | --- |
| *L. stricklandiae* | 1700–2000 | T4 |
| *L. Longisepala* | 750–1500 | T3, 6 |
| *L. sancta* | 1900–2100 | T6 |
| *L. lukwangulensis* | 1700–2500 | T6 |
| *L. giberroa* | 1200–3050 | T2, 3, 4, 6, 8 |
| *L. mildbraedii* | 1800–3050 | T7 |
| *L. deckenii* | 2400–4500 | T2 |
| *L. deckenii* subsp. *incipiens* | 2700–3000 | T2 |
| *L. burttii* | 3150–3800 | T2 |
| *L. burttii* subsp. *telmaticola* | 3000–3900 | T2 |
| *L. burtii* subsp. *meruensis* | 3150–3900 | T2 |
| *L. erinus* | 0–2450 | T4, 7, Z |
| *L. trullifolia* | 1000–2700 | T2, 3,6,7,8 |
| *L. trullifolia* subsp. *mino* | 1050–2200 | T7, 8 |
| *L. gilgii* | 1500–2500 | T6 |
| *L. graniticola* | 2100–2500. | T6 |
| *L. welwitschii* | 430–2740 | T4, 7, 8 |
| *L*. *fervens* subsp. *fervens* | 10–1850 | T1, 3, 4, 6, 7, 8, Z |
| *L*. *fervens* subsp. *recurvata* | 400–1500 | T1, 2, 5–7 |
| *L. uluginosa* | 1000–1800 | T4 |
| *L. sapinii* | 400–1050 | T6 |
| *L. chireensis* | 500–1250 | T4 |
| *L.morogorensis* | 700–1400 | T3 |
| *L. ritabeaniana* | 2000–2250 | T3 |
| *L. molleri* | 850–2500 | T6–8 |
| *L. rubescens* | 700–3000 | T6 |
| *L. heyneana* | 1000–1800 | T3 |
| *L. minutula* | 1200–3300 | T2, 7 |
| *L. adnexa* | 1000–1600 | T7 |
| *L. inconspicua* | 1000–2350 | T2, 4, 7, 8 |
| *L. duriprati* | 1500–3200 | T2 |
| *L. cymbalarioides* | 1500–3000 | T2, 3, 6 |
| *L. holstii* | 900–3000 | T2, 3, 5–7 |
| *L. goetzei* | 1000–3000 | T7, 8 |
| *L. ovina* | 1800–2500 | T7, 8 |
| *L. baumannii* | 700–2450 | T2–4, 6–8 |
| *L. angolensis* | 1600–2200 | T7 |
| *L. undzungwensis* | 1525–2400 | T4 |

Table S9. Lobelias and altitudinal distribution in the Uganda Flora regions (U1–4), a sub-flora of the *Flora of Tropical East Africa*.

| **Taxa name** | **Elevation in the region (m)** | **Early administrative Flora Regions** |
| --- | --- | --- |
| *L. giberroa* |  | U1–3 |
| *L. stuhlmannii* | 3000–4000 | U2 |
| *L. wollastonii* | 3300–4400 | U2 |
| *L. telekii* | 3000–4200 | U3 |
| *L. mildbraedii* | 1800–3050 | U2 |
| *L. aberdarica* | 1700–3500 | U3 |
| *L. bequaertii* | 3250–4100 | U2 |
| *L. gregoriana* subsp. *elgonensis* | 3400–4100 | U3 |
| *L. dissecta* | 1500–2250 | U1 |
| *L. flaccida* subsp. *granvikii* | 1200–3200 | U1, 3, 4 |
| *L. welwitschii* | 400–3200 | U1 |
| *L*. *fervens* subsp. *recurvata* | 400–1600 | U2–4 |
| *L. molleri* | 800–2400 | U2–4 |
| *L. rubescens* | 400–3000 | U2 |
| *L. minutula* | 1200–2350 | U2 |
| *L. inconspicua* | 1000–2350 | U4 |
| *L. duriprati* | 1500–3500 | U3 |
| *L. hartlaubii* | 500–1300 | U2 |
| *L. lindblomii* | 3000–4300 | U3 |

Table S10. Lobelias and altitudinal distribution in the Kenya Flora regions (K1-7), a sub-flora of the *Flora of Tropical East Africa*.

| **Taxa name** | **Elevation in the region (m)** | **Early administrative Flora Regions** |
| --- | --- | --- |
| *L. giberroa* | 1200–3050 | K1, 3–7 |
| *L. bambuseti* | 1800–3300 | K4 |
| *L. telekii* | 3000–5000 | K3, 4 |
| *L. aberdarica* | 1700–3550 | K3–6 |
| *L. gregoriana* | 3200–4500 | K4 |
| *L. gregoriana* subsp. *sattimae* | 3300–4000 | K4 |
| *L. gregorianai* subsp. *elgonensis* | 3400–4100 | K3 |
| *L. trullifolia* subsp. *trullifolia* | 1000–2750 | K7 |
| *L. flaccida* subsp. *granvikii* | 1200–3200 | K3, 5, 6 |
| *L. neumannii* | 1800–2800 | K2–4 |
| *L. welwitschii* | 2400–3200 | K3, 5 |
| *L. fervens* | 10–1850 | K1, 4, 7 |
| *L*. *fervens* subsp. *recurvata* | 400–1600 | K4 |
| *L. minutula* | 1200–4000 | K3, 4, 6 |
| *L. inconspicua* | 1200–2400 | K2–5 |
| *L. duriprati* | 1710–3600 | K3–6 |
| *L. holstii* | 1500–3500 | K1–7 |
| *L. lindblomii* | 3150–4300 | K3, 4 |
| *L. cheranganiensis* | 2500–3400 | K3 |
| *L. baumannii* | 2000–2400 | K4 |
| *L. angolensis* | 1700–2200 | K3 |

Table S11. Lobelias and distribution in Rwanda, a sub- flora of *Flora of Tropical East Africa*. Amfr = Afro-montane forest region, A-ar = Afro-alpine region, Gr = Grassland region, Wsr = Wetter savanna region

| **Taxa name** | **Elevation in the region (m)** | **Habitat & vegetation regions** |
| --- | --- | --- |
| *L. giberroa* | 1200–3050 | Upland forest edges (Amfr) |
| *L. stuhlmannii* | 3000–4000 | Erica and moorland (A-ar) |
| *L. wollastonii* | 3300–4400 | moorland (A-ar) |
| *L. petiolata* | 1900–2100 | Moist forest (Amfr) |
| *L. rubescens* | 700–3000 | Forest, wetlands banks (Amfr) |
| *L. minutula* | 1200–4000 | Moorland and Forest (A-ar, Amfr) |
| *L. holstii* | 1500–3500 | Disturbed moorland and forest (A-ar, Amfr, Gr, Wsr) |
| *L. hartlaubii* | 500–1300 | River banks and forest (Amfr) |

Table S12. Lobelias and distribution in Burundi, a sub-flora of *Flora of Tropical East Africa*. Amfr = Afro-montane forest region, A-ar = Afro-alpine region, Gr = Grassland region, Wsr = Wetter savanna region

| **Taxa name** | **Elevation in the region (m)** | **Habitat & vegetation regions** |
| --- | --- | --- |
| *L. giberroa* | 1200–3050 | Upland forest edges (Amfr) |
| *L. dissecta* | 1500–2250 | Open rocky area and forest open areas (Amfr) |
| *L*. *fervens* subsp. *recurvata* | 400–1600 | Marshy areas, Savanna, forest (Amfr) |
| *L. chireensis* | 500–1250 | Marshy muddy areas (Amfr, Wsr) |
| *L. molleri* | 800–2400 | Upland shady and moist places (Amfr, Wsr, Gr) |
| *L. rubescens* | 700–3000 | Forest, wetlands banks (Amfr) |
| *L. minutula* | 1200–4000 | Moorland and Forest (A-ar, Amfr) |
| *L. holstii* | 1500–3500 | Disturbed moorland and forest (A-ar, Amfr, Gr, Wsr) |
| *L. hartlaubii* | 500–1300 | River banks and forest (Amfr) |
